# Prevalence and Prognosis of Fever Symptoms, Hypo-, and Hyperthermia in Unselected Emergency Patients

**DOI:** 10.3390/jcm11010024

**Published:** 2021-12-22

**Authors:** Alexandra Malinovska, Liliana Malinovska, Christian H. Nickel, Roland Bingisser

**Affiliations:** 1 Department of Emergency Medicine, University Hospital Basel, 4031 Basel, Switzerland; christian.nickel@usb.ch (C.H.N.); roland.bingisser@usb.ch (R.B.); 2 Department of Emergency Medicine, Johns Hopkins University School of Medicine, Baltimore, MD 21205, USA; 3 Department of Epidemiology, Johns Hopkins University Bloomberg School of Public Health, Baltimore, MD 21205, USA; 4 Institute of Molecular Systems Biology, Department of Biology, ETH Zurich, 8093 Zurich, Switzerland; malinovska@imsb.biol.ethz.ch

**Keywords:** fever, temperature, hypothermia, hyperthermia, risk stratification, in-hospital mortality, symptoms, diagnostic, prognostic, emergency department

## Abstract

Assessments of history and body temperature are cornerstones of the diagnostic workup in all patients presenting to emergency departments (ED). Yet, the objective measurement of temperature and the subjective perception of fever can differ. This is a secondary exploratory analysis of a consecutive all-comer study, performed at an adult ED in Switzerland. Trained medical students interviewed all patients if fever was present. Altered temperature (>38.0 °C/<36.0 °C) measured at triage using an ear thermometer was used as the reference standard for diagnostic performance. In case of a disagreement between fever symptoms and altered temperature, discordance was noted. Outcome measures for case severity (acute morbidity, hospitalization, intensive care, and in-hospital mortality) were extracted from the electronic health records. Odds ratios (OR) for discordance between signs and symptoms and outcomes were calculated. Among 2183 patients, 325 patients reported fever symptoms. The sensitivity of fever symptoms as a test for altered temperature was 36.3%. Specificity was 91.5%. The negative predictive value was 84.1%, positive likelihood ratio was 4.2 and negative likelihood ratio was 0.7. The adjusted OR for discordance between fever symptoms and altered temperature was 1.71 (95% CI: 1.2–2.44) for acute morbidity, 1.56 (95% CI: 1.13–2.15) for hospitalization, and 1.12 (95% CI: 0.64–1.59) for intensive care. Unadjusted OR for mortality was 1.5 (95% CI: 0.69–3.25). Fever symptoms and altered temperature broadly overlap, but presentations can be stratified according to concordance between signs and symptoms. In case of discordance, the odds for acute morbidity and hospitalization are increased. Discordance may therefore be further investigated as a red flag for a serious outcome.

## 1. Introduction

Body temperature is routinely assessed, e.g., at presentation to emergency departments (ED). The early recognition of hyper- and hypothermia is crucial for public health reasons, risk stratification, and protocol-based care. Body temperature can be taken as a sign only, or as a starting point for a work-up. On the other hand, symptoms are also starting points for clinical evaluation. However, there can be an obvious difference between the sign (objective measurement of temperature) and the symptom (subjective perception of fever). Some patients might perceive fever (“feel feverish”), but their body temperature is within normal ranges. Conversely, patients not feeling feverish may have a body temperature out of range. While this issue is still under-investigated, some data show a good agreement between the symptoms of fever and the objective assessment of hyperthermia [1]; others demonstrate only medium to poor levels of agreement [2,3,4]. An even more intriguing question is the accordance between the fever symptoms and hypothermia—some data demonstrating a similar rate of “feverish” patients with documented hypo- and hyperthermia, respectively [5]. This lack of knowledge is enhanced by the fact that most published data are on young adults with low rates of comorbidity. There is neither evidence in unselected ED populations on the prevalence and prognosis of fever as a symptom, nor on the prevalence and prognosis of hypo- or hyperthermia. Several studies have shown that, in patients with suspected sepsis, hypothermic temperature was associated with poorer survival [6,7]. In all-comer intensive care units (ICU), patients’ hypothermia was associated with a poorer survival [8,9,10]. However, the role of hyperthermia is still debated, as data showing a survival benefit in hyperthermic patients [9,11] are offset by data demonstrating a higher mortality in hyperthermic patients [10,12]. Therefore, the first aim of this study was to assess the prevalence and prognosis of fever in an unselected ED population.

The question of whether the lack of a fever history and fever symptoms is predictive of normal body temperature is of interest, as most health authorities have endorsed test strategies based on respiratory symptoms and fever during the ongoing COVID-19 pandemic. Therefore, the second aim was to use the perception of fever (symptoms, such as “feeling feverish”) as a test in order to determine the test characteristics for the outcome “altered temperature”.

Further, we have previously shown that an “incoherent history” in patients with nonspecific complaints is predictive of serious outcomes [13]. The discordance between the patients’ information (considered to be subjective) and objective measurements by caregivers may indeed be a signal similar to a “red flag”. If symptoms do not correspond to the associated signs, physicians tend to ignore a part of the information, but this “physician filter” is associated with both higher rates of hospitalization and higher use of resources [14,15]. Therefore, the third aim was to assess discordance between fever as a symptom and altered body temperature as a sign: first, to gauge its prevalence, and second, to use discordance to stratify patient groups regarding outcomes.

## 2. Materials and Methods

### 2.1. Study Design and Setting

This study is a secondary exploratory analysis of a consecutive all-comer study. We acquired data for this prospective consecutive study for 6 weeks (From 21 October 2013 until 11 November 2013 and from 1 February 2015 until 23 February 2015). The study was performed at the emergency department (ED) of a 700-bed tertiary care hospital, where over 50,000 patients are seen per year. The local ethics committee approved the study (236/13, eknz).

### 2.2. Selection of Patients

All consecutive patients presenting to the ED were screened. Patients who did not give consent were not included. Pediatric, obstetric, and ophthalmology patients were treated at nearby facilities. Multiple presentations within the study period were excluded from the analysis.

### 2.3. Data Collection 

All patients presenting to the emergency department were screened by a member of the study team and an electronic health record (EHR) was opened. Patients were screened 24 h a day, 7 days a week. The study team consisted of trained medical students. They interviewed every patient at presentation about her/his/their symptoms, multiple answers were allowed. They used a questionnaire with 35 symptoms, but no additional questions were asked about duration, onset, and degree of fever. Specifically, the question “do you feel feverish or is this feeling the reason you presented to the ED?” was used.

The data were reviewed by an external institution (Health Care Research Institute, Zürich, Switzerland, owned by Swiss Post), digitalized, and anonymized.

### 2.4. Triage

A triage nurse or emergency physician triaged all patients according to the Emergency Severity Index (ESI). The German version of the ESI (version 4) was used [16]. The ESI categorizes patients into five levels based on acuity and expected resource consumption. 

### 2.5. Measurements and Outcomes

Temperature was measured during triage using an ear thermometer. According to the triage tool used (ESI), temperature was measured in patients who received life-saving interventions (ESI 1), were in high-risk situations (ESI 2), were considered to use more than one external resource (ESI 3), and with suggestive symptoms (ESI 4), but not in ESI 5 (see-and-treat patients). A normal temperature was defined according to the normal range of the NEWS (National Early Warning Score) criteria [17]: elevated temperature was defined as >38.0 °C, and “altered temperature” was defined as >38.0 °C or <36.1 °C. 

Information about demographics (age, gender), ESI triage level, disposition (hospitalization, and intensive care unit admission), and in-hospital mortality was extracted from the EHR. “Acute morbidity” is a novel outcome parameter, representing the patient’s acute need of medical intervention. Assessment of this parameter was performed using chart abstraction. This outcome parameter was validated in the cohort used in the study [18].

Only patients with full data sets were analyzed. We excluded patients with missing triage information or vital signs.

### 2.6. Stratifications

All presentations included were stratified according to two different principles: First, the presence of normal, elevated, or altered temperature, as described above.

Second, the concordance or discordance of perceived and measured fever:

- 2(A) Presentations with concordance of perceived fever and measured temperature:
  ○ A1: Fever symptoms perceived and temperature out of range;
  ○ A2: No fever symptoms perceived and temperature in range.
- 2(B) Presentations with discordance of perceived fever and measured temperature:
  ○ B1: Fever symptoms perceived, but temperature in range;
  ○ B2: No fever symptoms perceived, but temperature out of range.

### 2.7. Statistical Analyses

Descriptive statistics with frequencies and proportion or median and interquartile range for non-normally distributed data were computed. For group differences in binary or categorical data, we used Chi-squared or exact Fisher test when the expected values are low and *t*-test for continuous variables (with equal variance for age and unequal variance for temperature). For diagnostic test performance using the perception of fever as test and measured temperature as the reference standard, we calculated sensitivity, specificity, positive and negative predictive value, and positive and negative likelihood ratios with corresponding 95% confidential intervals. Using an univariable logistic regression with 20% jackknife cross validation, we calculated the diagnostic odds ratio. For the outcome association between concordance, discordance of the predictor variable and the different outcome variables, we used univariable and multivariable logistic regression models using a priori defined confounders (age, sex, and temperature category). Correlation analysis between variables was performed using Chi-squared test and Cramer’s V. A *p*-value < 0.05 was considered significant. All analyses were conducted using R version 4.0.5 (The R Foundation for Statistical Computing, Austria).

## 3. Results

### 3.1. Characteristics of Study Subjects

**Figure 1:**
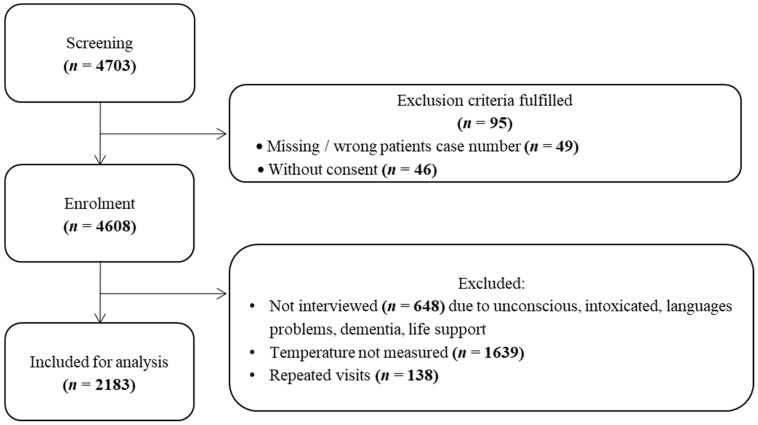
Patient enrollment chart.

In total, 5634 presentations were recorded during the study periods, 4608 presentations were formally screened, and 2183 presentations were included (Figure 1). Exclusion criteria were lack of data, age under 18, and lack of consent. Patients who had made repeated visits were excluded. The median age was 56 years; 40% of all presentations were older than 65 years. Gender was almost equally distributed (52% female), and 1522 cases were from northern or central Europe. Almost half of all presentations (1071) were assigned to ESI 3 (Table 1).

#### 3.1.1. Patient Strata According to Temperature

Elevated body temperature was detected in 252 (11.5%) presentations and reduced body temperature in 218 (10.0%) of all included presentations (Table 2)

#### 3.1.2. Patient Strata According to Symptoms

In total, 325 (14.9%) of all cases reported a subjective feeling of fever at presentation. Among all cases with elevated (>38.0 C) temperature, 67.5% felt feverish. Among all cases with reduced (<36.1 C) temperature, 3.7% felt feverish.

#### 3.1.3. Patient Strata According to Concordance of Perceived and Measured Temperature

(A) We found a concordance of the perceived and measured temperatures in 1744 cases. A total of 178 cases had perceived fever symptoms and altered temperature was found (A1). This included 170 cases with a temperature >38.0 °C and eight cases with a temperature <36.1 °C. The median age was 54.5 years. In 1566 presentations (71.7% of all presentations), no fever symptoms and normal temperature were reported; the median age of patients was 55 years (Table 3).

(B) A discordance of perceived and measured temperatures was found in 439 patients. In 147 cases, fever symptoms were perceived, but no fever was found (B1). These patients were younger (median age—47 years) than patients with concordance. Additionally, 292 patients not reporting fever (15.7% of cases did not report fever), a body temperature out of range was detected (B2). Altered temperature was elevated (>38.0 °C) in 82 cases and reduced (<36.1 °C) in 210 cases. Patients were older (median age—66 years) compared to patients with a concordance of perceived and measured temperatures (Table 3).

### 3.2. Diagnostic Test Performance

The sensitivity of fever history as a test for temperature out of range was 36.4% (95% CI: 32.1–40.8%). Specificity was 91.5% (95% CI: 90.1–92.7%), and the negative predictive value was 84.1% (95% CI: 82.4–85.7%) with a prevalence of 21.3% (Table 4). 

The diagnostic odds ratio for the perception of fever as a test and temperature measurement as the reference standard using an 20% jackknife cross-validated regression model showed an AUC of 0.62. 

### 3.3. Association to Outcome Measures

Predefined outcomes were acute morbidity, hospitalization, admission to intensive care, and in-hospital mortality. In total, 1021 cases are considered acute morbid, 905 presentations (41.5%) were hospitalized, and 6.8% were admitted to the intensive care unit and 1.5% died in hospital. These results are generally comparable to the entire all-comer cohort (33.5% hospitalized, 6.5% received intensive care, and 1.6% died in hospital).

Cases not reporting fever while having an altered temperature had the highest rates of intensive care (10.3%) and in-hospital mortality (3.1%) (Table 3). 

The type of concordance respective discordance (A1, A2, B1, B2) showed a strong correlation with the temperature category (Chi-square test *p*-value < 0.0001, Cramer’s V: 0.846); thus, we used patients’ strata (A vs. B) to assess the association with relevant prognostic outcome measures. 

The crude and adjusted odds ratio of discordance of fever perception and measured temperature for all outcomes are shown in Table 5. The adjustment of the interaction between concordance and temperature only showed significantly different results for the outcome “acute morbidity”. 

## 4. Discussion

This study showed a high prevalence of fever in our ED all-comer population. Moreover, the outcomes between fever patients and the entire ED cohort are comparable. Stratification according to body temperature showed that, in patients with hypothermia, all outcomes were worse compared to patients with normo- or hyperthermia. A similar observation was reported in ED patients with bacterial infection, where a low temperature on arrival was associated with a higher risk of death [19]. 

If fever symptoms were to be taken as a test for predicting an out-of-range body temperature, sensitivity and specificity were too low to be useful. Furthermore, in patients without a perception of fever, a body temperature out of range could not be excluded due to a negative LR of −0.6. Taken together, neither a positive nor a negative history for fever symptoms can replace temperature measurements. On the other hand, the discordance between fever history and altered temperature may be interpreted as a meaningful signal. First, the prevalence of discordance was high. Second, patient characteristics were different, as these patients were generally older. Third, discordance between fever signs and symptoms can be used for prognosis. While concordant patients (A1: reporting fever symptoms in concordance with altered temperature, and A2: reporting no fever symptoms in concordance with normal temperature) had outcomes comparable to the entire ED cohort, patients with a discordance of signs and symptoms carried a worse prognosis. Most interesting, stratum B2 (altered temperature without fever symptoms) had the worst prognosis among all groups. Conversely, stratum B1 (normal temperature, but reporting fever symptoms) had a better prognosis. This is a new finding and merits discussion. The possible explanations of these findings are cultural differences regarding the perception and description of fever symptoms, the effect of old age and hypothermia [20], and cognitive issues in highly morbid patients [21]. Particularly in sepsis and COVID-19, the perception of fever may be impaired, and hyperthermia might be missing at an early stage [22,23]. Such patients may be tagged with an “incoherent history” [13] or delirium that often goes unnoticed at presentation and during the early hours of ED work-up [24]. Further, it is noteworthy that the vast majority of hypothermic patients in our cohort did not report on subjective feelings of fever. This could be attributed to an older age or altered physiology in hypothermic patients, or the absence of typical fever symptoms, such as shivering when temperature set points are offset.

### Limitation

The generalizability of our findings might be limited due to two main reasons: First, we conducted a single-center study. However, our study was the largest study performed and we used an unselected ED population. Second, the evaluation of diagnostic performance can be driven by the prevalence of the condition tested by the reference standards. Our data were collected during autumn and winter, where higher rates of respiratory infections might increase the fever prevalence. Therefore, the results might be biased by seasonal effects. 

Further, data collection focused on quantitative assessments, using questionnaires about symptoms with a binary yes vs. no answer. The question “did you or do you feel feverish?” might be interpreted differently from a patients’ or caregiver’s perspective. Patients might overly focus on the typical shivering due to rising temperature or “feeling hot” due to a persistent high body temperature. Qualitative research on the interpretation of the patient’s perceptive of fever could improve our understanding of the topic. Additionally, a more comprehensive medical history might be needed to better understand group differences. For example, we did not evaluate the self-use of antipyretic medication before ED presentation, which might be an explanation for the discordance when patients are reporting fever while their body temperature was normal. To evaluate the prognostic value of the concordance of fever symptoms with body temperature, we took mortality and intensive care as “hard” outcomes. While these outcomes are generally undisputed, they have a low likelihood in unselected ED cohorts. Therefore, we also used hospitalization and “acute morbidity” as outcomes. While hospitalization as an outcome may be criticized due to sociodemographic influences and high variance between health-care systems, acute morbidity is a newly validated outcome that has not been widely used.

## 5. Conclusions

We found a high prevalence of fever symptoms and a high prevalence of hypo- and hyperthermia. Subjective symptoms and objective signs broadly overlap, but presentations can be stratified according to concordance or discordance between the sign and symptom of fever. In case of discordance, particularly in hypothermia, caution is warranted, as this may be an early red flag for a serious outcome, such as intensive care or short-term mortality.

## Figures and Tables

**Table 1:** Baseline characteristics and outcome measures of fever history.

	Total	No Fever Symptoms	Fever Symptoms	*p*-Value
	*n* = 2183	*n* = 1858	*n* = 325	
Age, median (IQR) (years)	56.0 (37.0; 75.0)	57.0 (37.0; 76.0)	53.0 (33.0; 70.0)	0.001
Female Sex, n (%)	1124 (51.5%)	967 (52.0%)	157 (48.3%)	0.23
Temperature, median (IQR) (°C)	36.9 (36.5; 37.5)	36.8 (36.4; 37.2)	38.1 (37.3; 38.8)	<0.001
ESI Triage Category, n (%)				0.45
1	29 (1.3%)	28 (1.5%)	1 (0.3%)	
2	588 (27.0%)	502 (27.1%)	86 (26.5%)	
3	1071 (49.1%)	912 (49.2%)	159 (48.9%)	
4	470 (21.6%)	394 (21.2%)	76 (23.4%)	
5	22 (1.0%)	19 (1.0%)	3 (0.9%)	
Missing	3	3 (0.2%)	0 (0%)	
European, n (%)	1522 (69.9%)	1332 (71.9%)	190 (58.5%)	<0.001
Acute Morbidity, n (%)	1021 (46.8%)	850 (45.7%)	171 (52.6%)	0.03
Hospitalization, n (%)	905 (41.5%)	744 (40.0%)	161 (49.5%)	0.002
Intensive Care Admission, n (%)	149 (6.8%)	132 (7.1%)	17 (5.2%)	0.26
In-hospital Mortality, n (%)	33 (1.5%)	32 (1.7%)	1 (0.3%)	0.05

Abbreviation: ESI: emergency severity index; IQR: interquartile range; n: number. *p*-values based on Chi-squared or exact Fisher test when the expected values are low. *t*-test for continuous variables (age with equal variance, temperature with unequal variance).

**Table 2:** Baseline characteristics and outcome measures by temperature categories.

	Normal Temperature	High Temperature	Low Temperature	*p*-Value
	*n* = 1713	*n* = 252	*n* = 218	
Age, median (IQR) (years)	55.0 (35.0; 74.0)	59.0 (35.0; 77.0)	65.0 (44.2; 77.0)	0.002
Female Sex, n (%)	919 (53.6%)	108 (42.9%)	97 (44.5%)	0.001
Temperature, median (IQR) (°C)	36.9 (36.6; 37.3)	38.7 (38.3; 39.1)	35.9 (35.5; 36.0)	<0.001
ESI Triage Category, n (%)				
1	17 (1.0%)	3 (1.2%)	9 (4.1%)	
2	429 (25.1%)	87 (34.5%)	72 (33.0%)	
3	857 (50.1%)	121 (48.0%)	93 (42.7%)	
4	390 (22.8%)	39 (15.5%)	41 (18.8%)	
5	17 (1.0%)	2 (0.8%)	3 (1.4%)	
Missing	3	0	0	
European, n (%)	524 (30.7%)	80 (31.7%)	51 (23.4%)	0.072
Acute Morbidity, n (%)	762 (44.5%)	148 (58.7%)	111 (50.9%)	<0.001
Hospitalization, n (%)	666 (38.9%)	148 (58.7%)	91 (41.7%)	<0.001
Intensive Care Admission, n (%)	109 (6.4%)	19 (7.5%)	21 (9.6%)	0.176
In-hospital Mortality, n (%)	23 (1.3%)	2 (0.8%)	8 (3.7%)	0.033

Normal temperature: >38.0 °C or <36.1 °C; high temperature: ≥38.0 °C; low temperature: ≤36.0 °C. Abbreviation: ESI: emergency severity index, IQR: interquartile range, n: number; *p*-values based on Chi-squared or exact Fisher test when the expected values are low. *t*-test for continuous variables (age with equal variance, temperature with unequal variance).

**Table 3:** Baseline characteristics and outcome measures by classification between concordance and discordance of perceived fever and measured temperature.

	Concordance		Discordance		*p*-Value
Perceived Fever	No	Yes	No	Yes	
Temperature	Normal	Altered	Altered	Normal	
Number	*n* = 1566	*n* = 178	*n* = 292	*n* = 147	
Age, median (IQR) (years)	55.0 (36.0; 75.0)	54.5 (32.2; 72.0)	66.0 (44.8; 80.0)	47.0 (33.0; 66.5)	<0.001
Female Sex, n (%)	839 (53.6%)	77 (43.3%)	128 (43.8%)	80 (54.4%)	
Temperature, median (IQR) (°C)	36.9 (36.5; 37.2)	38.8 (38.3; 39.1)	36.0 (35.7; 38.1)	37.3 (36.8; 37.8)	<0.001
ESI Triage Category, n (%)					
1	17 (1.09%)	1 (0.56%)	11 (3.77%)	0 (0.00%)	
2	402 (25.7%)	59 (33.1%)	100 (34.2%)	27 (18.4%)	
3	781 (50.0%)	83 (46.6%)	131 (44.9%)	76 (51.7%)	
4	347 (22.2%)	33 (18.5%)	47 (16.1%)	43 (29.3%)	
5	16 (1.02%)	2 (1.12%)	3 (1.03%)	1 (0.68%)	
Missing	3	0	3	0	
European, n (%)	1101 (70.6%)	108 (60.7%)	231 (79.1%)	82 (55.8%)	<0.001
Acute Morbidity, n (%)	685 (43.7%)	94 (52.8%)	165 (56.5%)	77 (52.4%)	<0.001
Hospitalization, n (%)	601 (38.4%)	96 (53.9%)	143 (49.0%)	65 (44.2%)	<0.001
Intensive Care Admission, n (%)	102 (6.51%)	10 (5.62%)	30 (10.3%)	7 (4.76%)	0.07
In-hospital Mortality, n (%)	23 (1.47%)	1 (0.56%)	9 (3.08%)	0 (0.00%)	0.06

Abbreviation: ESI: emergency severity index; IQR: interquartile range; n: number. *p*-values based on Chi-squared or exact Fisher test when the expected values are low. *t*-test for continuous variables (age with equal variance, temperature with unequal variance).

**Table 4:** Binary classifiers for fever history as test for altered temperature.

Performance Test of Patients’ History Metric	Value	95% Confidence Interval
fever prevalence	21.3	
sensitivity	36.4%	32.1–40.8%
specificity	91.5%	90.1–92.7
positive predictive value	53.7%	48.2–59.1
negative predictive value	84.1%	82.4–85.7
positive likelihood ratio	4.2	3.5–5.2
negative likelihood ratio	0.7	0.6–0.7

**Table 5:** Crude and adjusted logistic regression of different case severity outcomes on discordance of perceived fever and measured temperature.

	Discordance of Perceived Fever and Measurement					
	Crude			Adjusted ^†^		
Outcome	Odds Ratio	95% CI	*p*-Value	Odds Ratio	95% CI	*p*-Value
Acute Morbidity	1.52	1.23–1.88	<0.001	1.71	1.2–2.44	0.003
Hospitalization	1.35	1.1–1.67	0.005	1.56	1.13–2.15	0.007
Intensive Care Admission	1.34	0.91–1.98	0.14	1.12	0.64–1.96	0.69
In-hospital-Mortality	1.5	0.69–3.25	0.30	--	--	--

^†^ Adjusted for confounding variables (age, sex, temperature categories) in hospitalization, intensive care admission, in-hospital mortality. Adjusted for confounding variables (age, sex, temperature categories, interaction agreement and temperature categories) in acute morbidity. No adjusted odds ratio was calculated for mortality due to small number of outcomes. Abbreviations: CI: confidence interval.

## Data Availability

The data presented in this study are available on request from the corresponding author.
